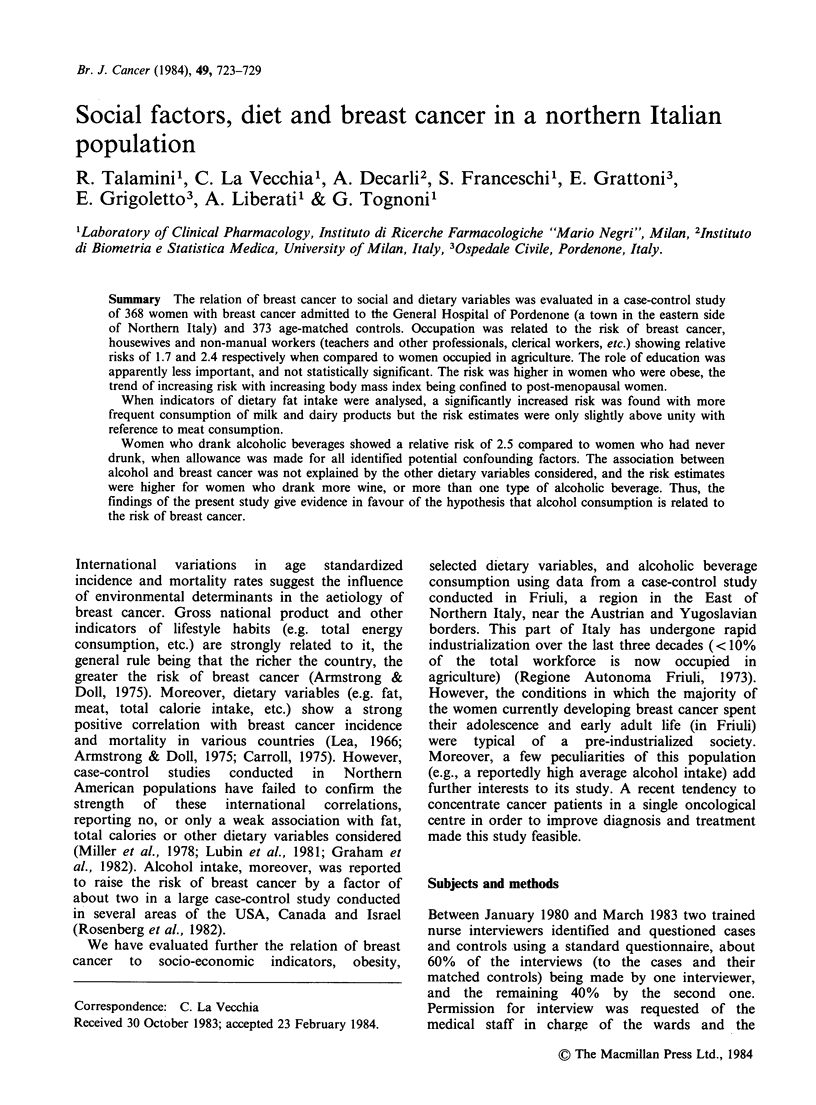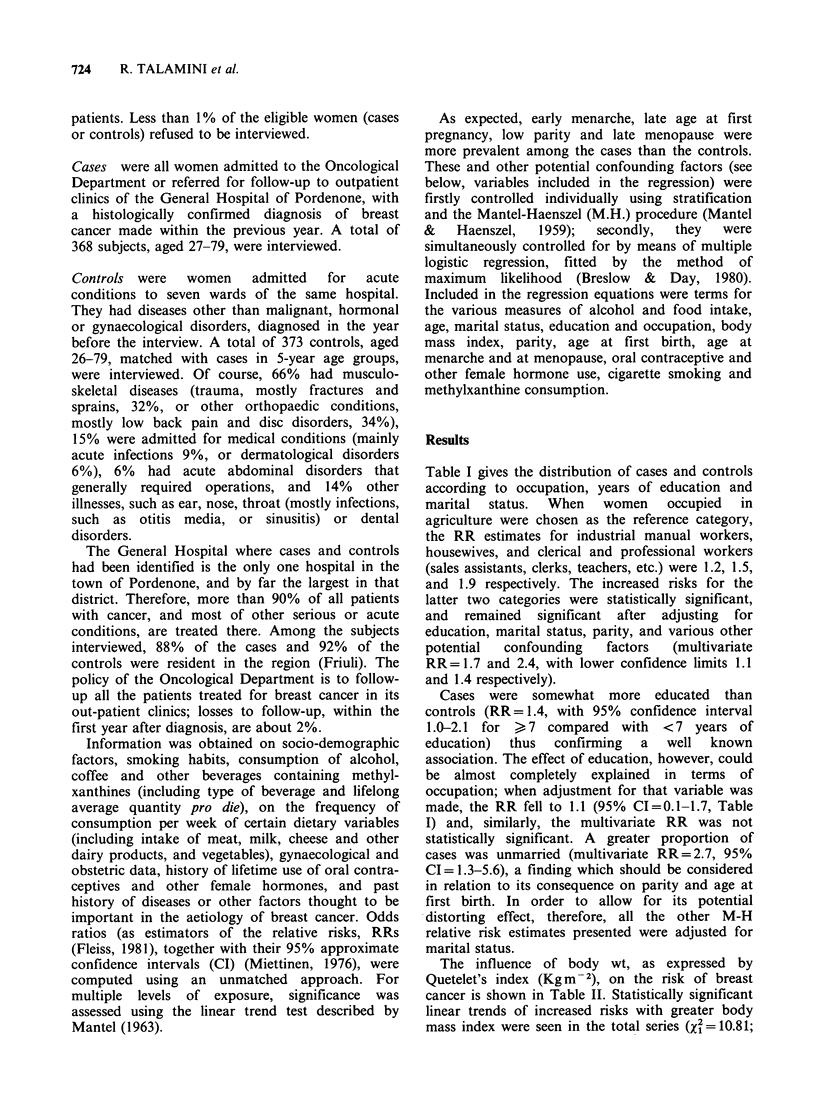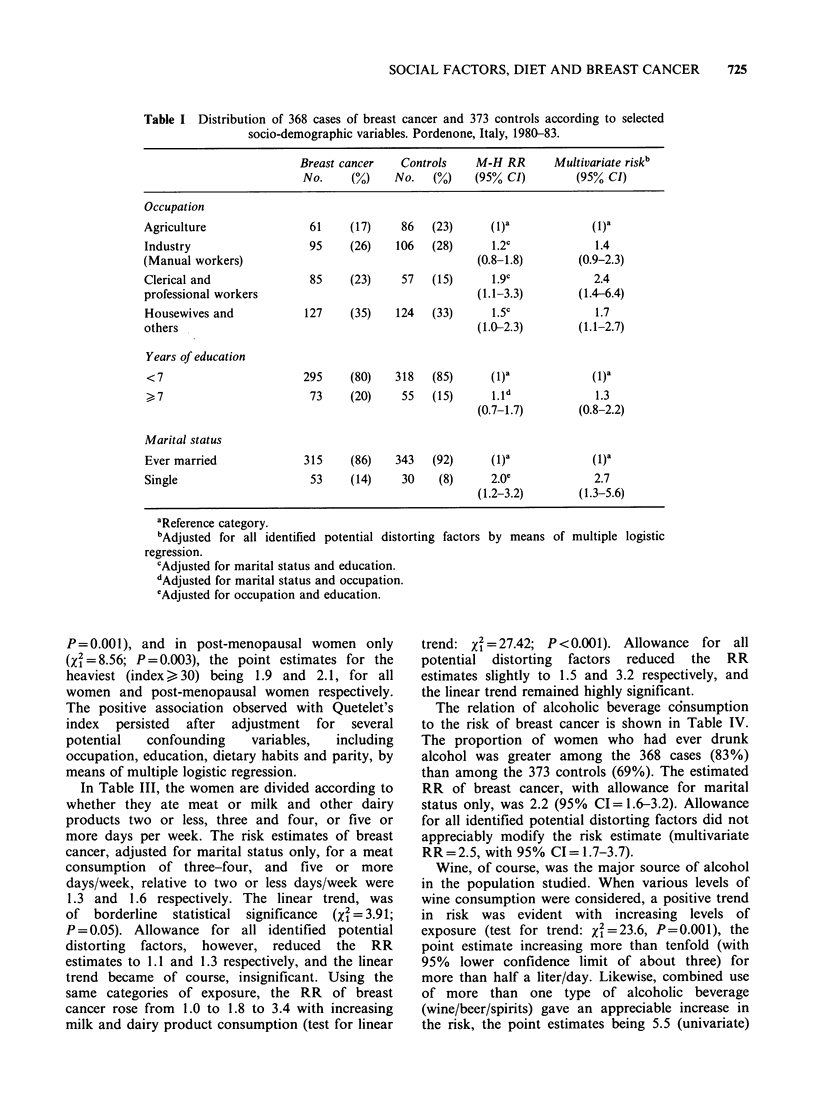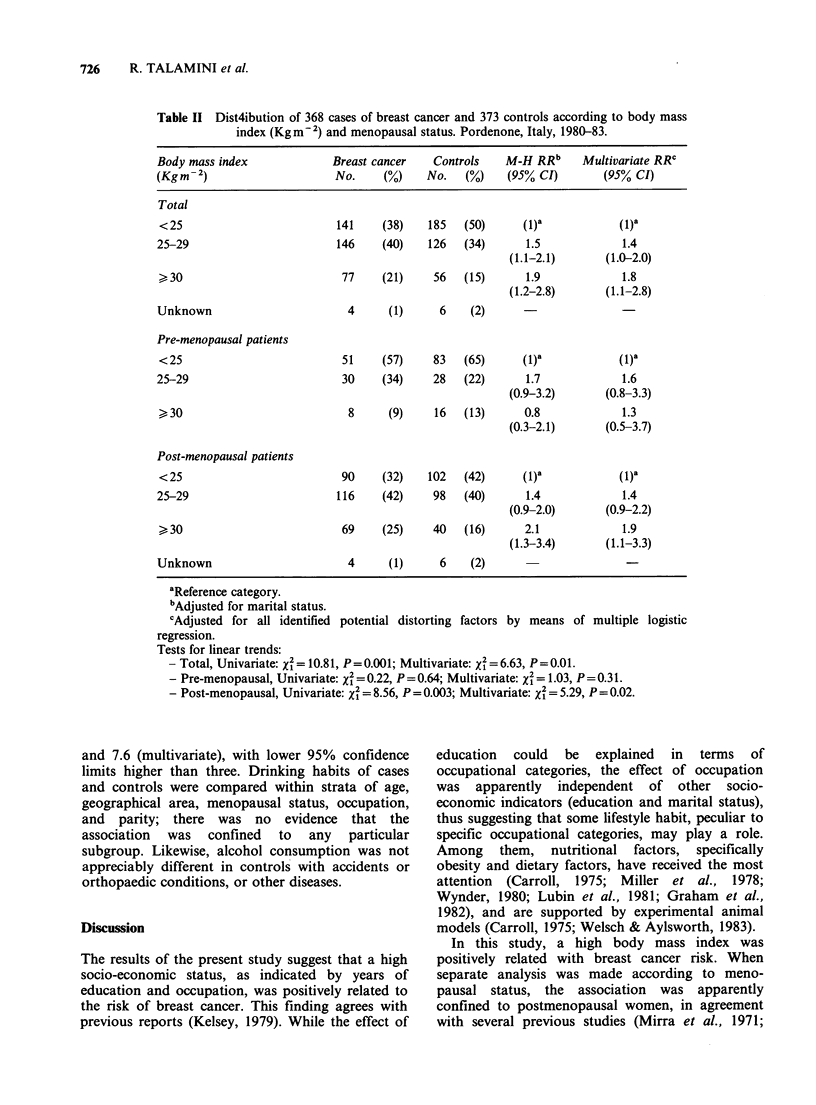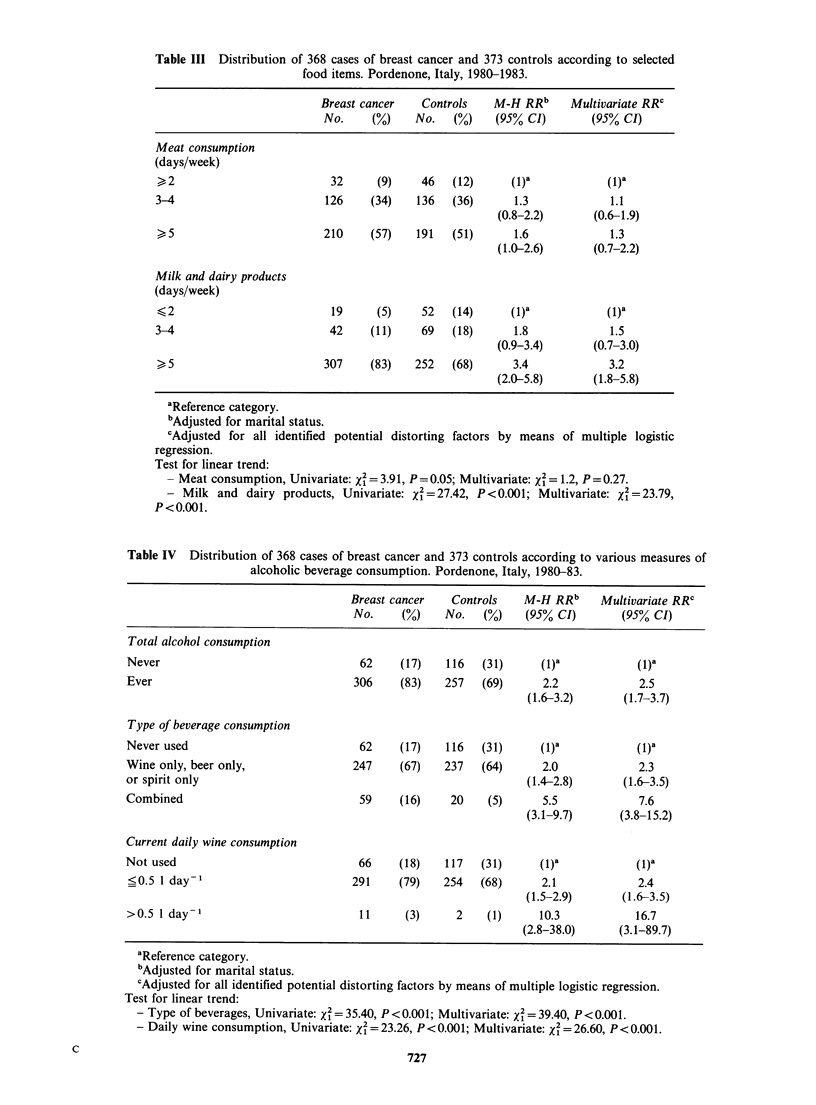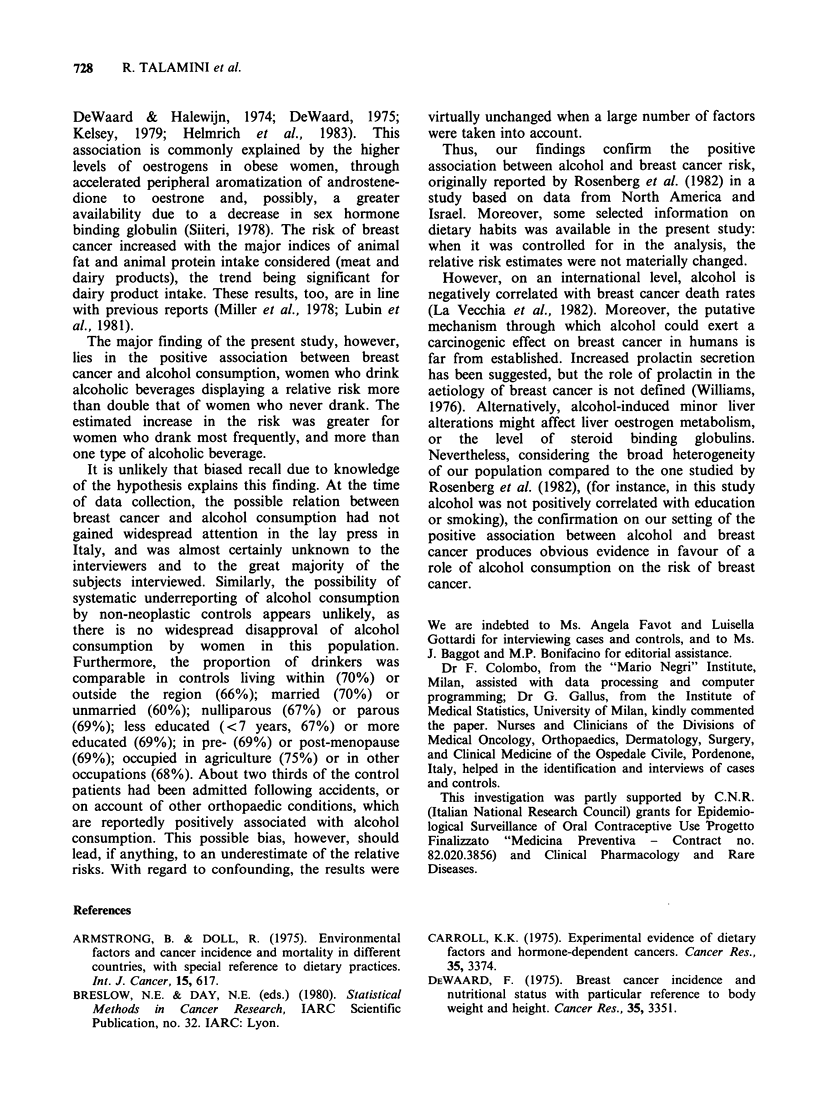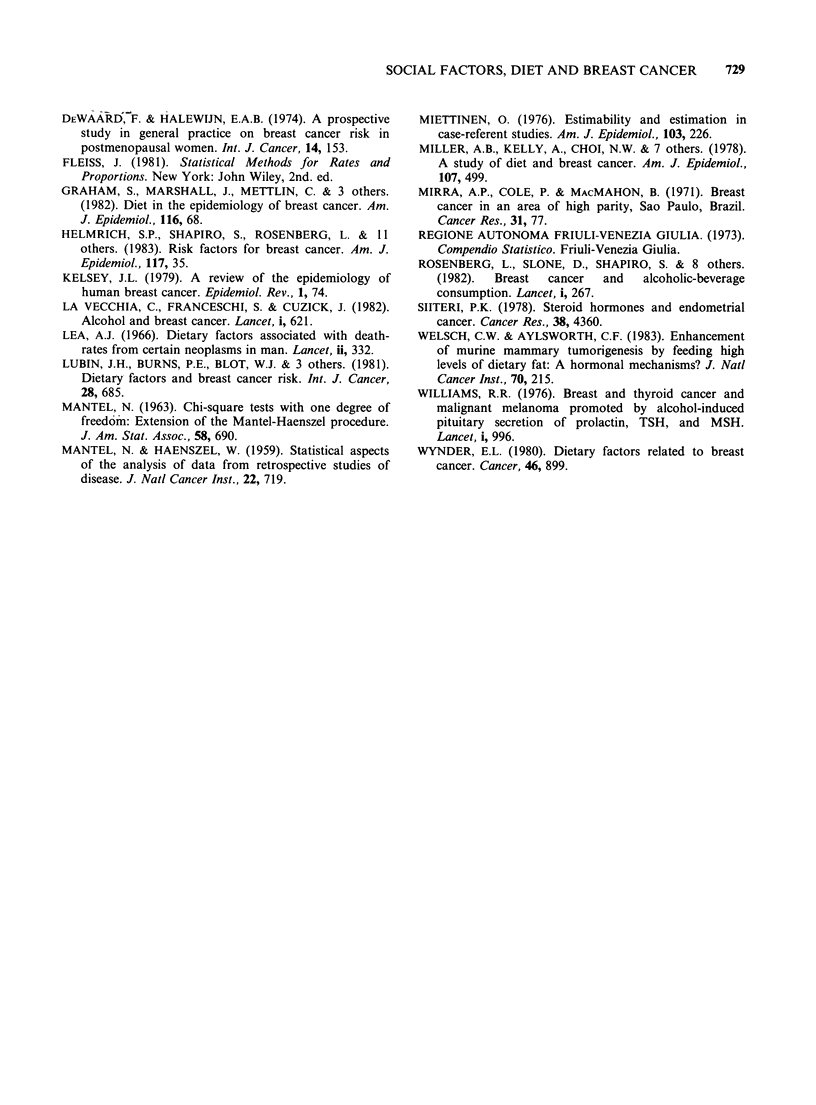# Social factors, diet and breast cancer in a northern Italian population.

**DOI:** 10.1038/bjc.1984.114

**Published:** 1984-06

**Authors:** R. Talamini, C. La Vecchia, A. Decarli, S. Franceschi, E. Grattoni, E. Grigoletto, A. Liberati, G. Tognoni

## Abstract

The relation of breast cancer to social and dietary variables was evaluated in a case-control study of 368 women with breast cancer admitted to the General Hospital of Pordenone (a town in the eastern side of Northern Italy) and 373 age-matched controls. Occupation was related to the risk of breast cancer, housewives and non-manual workers (teachers and other professionals, clerical workers, etc.) showing relative risks of 1.7 and 2.4 respectively when compared to women occupied in agriculture. The role of education was apparently less important, and not statistically significant. The risk was higher in women who were obese, the trend of increasing risk with increasing body mass index being confined to post-menopausal women. When indicators of dietary fat intake were analysed, a significantly increased risk was found with more frequent consumption of milk and dairy products but the risk estimates were only slightly above unity with reference to meat consumption. Women who drank alcoholic beverages showed a relative risk of 2.5 compared to women who had never drunk, when allowance was made for all identified potential confounding factors. The association between alcohol and breast cancer was not explained by the other dietary variables considered, and the risk estimates were higher for women who drank more wine, or more than one type of alcoholic beverage. Thus, the findings of the present study give evidence in favour of the hypothesis that alcohol consumption is related to the risk of breast cancer.


*Br. J. Cancer* (1984), **49**, 723–729

### Social factors, diet and breast cancer in a northern Italian population

R. Talamini[1], C. La Vecchia[1], A. Decarli[2], S. Franceschi[1], E. Grattoni[3], E. Grigoletto[3], A. Liberati[1] & G. Tognoni[1]

[1]*Laboratory of Clinical Pharmacology, Instituto di Ricerche Farmacologiche "Mario Negri", Milan,* [2]*Instituto di Biometria e Statistica Medica, University of Milan, Italy,* [3]*Ospedale Civile, Pordenone, Italy.*

**Summary** The relation of breast cancer to social and dietary variables was evaluated in a case-control study of 368 women with breast cancer admitted to the General Hospital of Pordenone (a town in the eastern side of Northern Italy) and 373 age-matched controls. Occupation was related to the risk of breast cancer, housewives and non-manual workers (teachers and other professionals, clerical workers, *etc.*) showing relative risks of 1.7 and 2.4 respectively when compared to women occupied in agriculture. The role of education was apparently less important, and not statistically significant. The risk was higher in women who were obese, the trend of increasing risk with increasing body mass index being confined to post-menopausal women.

When indicators of dietary fat intake were analysed, a significantly increased risk was found with more frequent consumption of milk and dairy products but the risk estimates were only slightly above unity with reference to meat consumption.

Women who drank alcoholic beverages showed a relative risk of 2.5 compared to women who had never drunk, when allowance was made for all identified potential confounding factors. The association between alcohol and breast cancer was not explained by the other dietary variables considered, and the risk estimates were higher for women who drank more wine, or more than one type of alcoholic beverage. Thus, the findings of the present study give evidence in favour of the hypothesis that alcohol consumption is related to the risk of breast cancer.

International variations in age standardized incidence and mortality rates suggest the influence of environmental determinants in the aetiology of breast cancer. Gross national product and other indicators of lifestyle habits (e.g. total energy consumption, etc.) are strongly related to it, the general rule being that the richer the country, the greater the risk of breast cancer (Armstrong & Doll, 1975). Moreover, dietary variables (e.g. fat, meat, total calorie intake, etc.) show a strong positive correlation with breast cancer incidence and mortality in various countries (Lea, 1966; Armstrong & Doll, 1975; Carroll, 1975). However, case-control studies conducted in Northern American populations have failed to confirm the strength of these international correlations, reporting no, or only a weak association with fat, total calories or other dietary variables considered (Miller *et al.*, 1978; Lubin *et al.*, 1981; Graham *et al.*, 1982). Alcohol intake, moreover, was reported to raise the risk of breast cancer by a factor of about two in a large case-control study conducted in several areas of the USA, Canada and Israel (Rosenberg *et al.*, 1982).

We have evaluated further the relation of breast cancer to socio-economic indicators, obesity, selected dietary variables, and alcoholic beverage consumption using data from a case-control study conducted in Friuli, a region in the East of Northern Italy, near the Austrian and Yugoslavian borders. This part of Italy has undergone rapid industrialization over the last three decades (<10% of the total workforce is now occupied in agriculture) (Regione Autonoma Friuli, 1973). However, the conditions in which the majority of the women currently developing breast cancer spent their adolescence and early adult life (in Friuli) were typical of a pre-industrialized society. Moreover, a few peculiarities of this population (e.g., a reportedly high average alcohol intake) add further interests to its study. A recent tendency to concentrate cancer patients in a single oncological centre in order to improve diagnosis and treatment made this study feasible.

### Subjects and methods

Between January 1980 and March 1983 two trained nurse interviewers identified and questioned cases and controls using a standard questionnaire, about 60% of the interviews (to the cases and their matched controls) being made by one interviewer, and the remaining 40% by the second one. Permission for interview was requested of the medical staff in charge of the wards and the

Correspondence: C. La Vecchia
Received 30 October 1983; accepted 23 February 1984.

© The Macmillan Press Ltd., 1984

patients. Less than 1% of the eligible women (cases or controls) refused to be interviewed.

*Cases* were all women admitted to the Oncological Department or referred for follow-up to outpatient clinics of the General Hospital of Pordenone, with a histologically confirmed diagnosis of breast cancer made within the previous year. A total of 368 subjects, aged 27–79, were interviewed.

*Controls* were women admitted for acute conditions to seven wards of the same hospital. They had diseases other than malignant, hormonal or gynaecological disorders, diagnosed in the year before the interview. A total of 373 controls, aged 26–79, matched with cases in 5-year age groups, were interviewed. Of course, 66% had musculo-skeletal diseases (trauma, mostly fractures and sprains, 32%, or other orthopaedic conditions, mostly low back pain and disc disorders, 34%), 15% were admitted for medical conditions (mainly acute infections 9%, or dermatological disorders 6%), 6% had acute abdominal disorders that generally required operations, and 14% other illnesses, such as ear, nose, throat (mostly infections, such as otitis media, or sinusitis) or dental disorders.

The General Hospital where cases and controls had been identified is the only one hospital in the town of Pordenone, and by far the largest in that district. Therefore, more than 90% of all patients with cancer, and most of other serious or acute conditions, are treated there. Among the subjects interviewed, 88% of the cases and 92% of the controls were resident in the region (Friuli). The policy of the Oncological Department is to follow-up all the patients treated for breast cancer in its out-patient clinics; losses to follow-up, within the first year after diagnosis, are about 2%.

Information was obtained on socio-demographic factors, smoking habits, consumption of alcohol, coffee and other beverages containing methyl-xanthines (including type of beverage and lifelong average quantity *pro die*), on the frequency of consumption per week of certain dietary variables (including intake of meat, milk, cheese and other dairy products, and vegetables), gynaecological and obstetric data, history of lifetime use of oral contraceptives and other female hormones, and past history of diseases or other factors thought to be important in the aetiology of breast cancer. Odds ratios (as estimators of the relative risks, RRs (Fleiss, 1981), together with their 95% approximate confidence intervals (CI) (Miettinen, 1976), were computed using an unmatched approach. For multiple levels of exposure, significance was assessed using the linear trend test described by Mantel (1963).

As expected, early menarche, late age at first pregnancy, low parity and late menopause were more prevalent among the cases than the controls. These and other potential confounding factors (see below, variables included in the regression) were firstly controlled individually using stratification and the Mantel-Haenszel (M.H.) procedure (Mantel & Haenszel, 1959); secondly, they were simultaneously controlled for by means of multiple logistic regression, fitted by the method of maximum likelihood (Breslow & Day, 1980). Included in the regression equations were terms for the various measures of alcohol and food intake, age, marital status, education and occupation, body mass index, parity, age at first birth, age at menarche and at menopause, oral contraceptive and other female hormone use, cigarette smoking and methylxanthine consumption.

### Results

Table I gives the distribution of cases and controls according to occupation, years of education and marital status. When women occupied in agriculture were chosen as the reference category, the RR estimates for industrial manual workers, housewives, and clerical and professional workers (sales assistants, clerks, teachers, etc.) were 1.2, 1.5, and 1.9 respectively. The increased risks for the latter two categories were statistically significant, and remained significant after adjusting for education, marital status, parity, and various other potential confounding factors (multivariate RR = 1.7 and 2.4, with lower confidence limits 1.1 and 1.4 respectively).

Cases were somewhat more educated than controls (RR = 1.4, with 95% confidence interval 1.0–2.1 for $\geqslant 7$ compared with $<7$ years of education) thus confirming a well known association. The effect of education, however, could be almost completely explained in terms of occupation; when adjustment for that variable was made, the RR fell to 1.1 (95% CI = 0.1–1.7, Table I) and, similarly, the multivariate RR was not statistically significant. A greater proportion of cases was unmarried (multivariate RR = 2.7, 95% CI = 1.3–5.6), a finding which should be considered in relation to its consequence on parity and age at first birth. In order to allow for its potential distorting effect, therefore, all the other M-H relative risk estimates presented were adjusted for marital status.

The influence of body wt, as expressed by Quetelet's index ($\mathrm{Kg\,m^{-2}}$), on the risk of breast cancer is shown in Table II. Statistically significant linear trends of increased risks with greater body mass index were seen in the total series ($\chi_1^2 = 10.81$;

**Table I** Distribution of 368 cases of breast cancer and 373 controls according to selected socio-demographic variables. Pordenone, Italy, 1980–83.

| | *Breast cancer* No. | (%) | *Controls* No. | (%) | *M-H RR* (95% *CI*) | *Multivariate risk*[b] (95% *CI*) |
|---|---|---|---|---|---|---|
| *Occupation* | | | | | | |
| Agriculture | 61 | (17) | 86 | (23) | (1)[a] | (1)[a] |
| Industry (Manual workers) | 95 | (26) | 106 | (28) | 1.2[c] (0.8–1.8) | 1.4 (0.9–2.3) |
| Clerical and professional workers | 85 | (23) | 57 | (15) | 1.9[c] (1.1–3.3) | 2.4 (1.4–6.4) |
| Housewives and others | 127 | (35) | 124 | (33) | 1.5[c] (1.0–2.3) | 1.7 (1.1–2.7) |
| *Years of education* | | | | | | |
| <7 | 295 | (80) | 318 | (85) | (1)[a] | (1)[a] |
| ≥7 | 73 | (20) | 55 | (15) | 1.1[d] (0.7–1.7) | 1.3 (0.8–2.2) |
| *Marital status* | | | | | | |
| Ever married | 315 | (86) | 343 | (92) | (1)[a] | (1)[a] |
| Single | 53 | (14) | 30 | (8) | 2.0[e] (1.2–3.2) | 2.7 (1.3–5.6) |

[a]Reference category.
[b]Adjusted for all identified potential distorting factors by means of multiple logistic regression.
[c]Adjusted for marital status and education.
[d]Adjusted for marital status and occupation.
[e]Adjusted for occupation and education.

$P=0.001$), and in post-menopausal women only ($\chi_1^2=8.56$; $P=0.003$), the point estimates for the heaviest (index≥30) being 1.9 and 2.1, for all women and post-menopausal women respectively. The positive association observed with Quetelet's index persisted after adjustment for several potential confounding variables, including occupation, education, dietary habits and parity, by means of multiple logistic regression.

In Table III, the women are divided according to whether they ate meat or milk and other dairy products two or less, three and four, or five or more days per week. The risk estimates of breast cancer, adjusted for marital status only, for a meat consumption of three–four, and five or more days/week, relative to two or less days/week were 1.3 and 1.6 respectively. The linear trend, was of borderline statistical significance ($\chi_1^2=3.91$; $P=0.05$). Allowance for all identified potential distorting factors, however, reduced the RR estimates to 1.1 and 1.3 respectively, and the linear trend became of course, insignificant. Using the same categories of exposure, the RR of breast cancer rose from 1.0 to 1.8 to 3.4 with increasing milk and dairy product consumption (test for linear trend: $\chi_1^2=27.42$; $P<0.001$). Allowance for all potential distorting factors reduced the RR estimates slightly to 1.5 and 3.2 respectively, and the linear trend remained highly significant.

The relation of alcoholic beverage consumption to the risk of breast cancer is shown in Table IV. The proportion of women who had ever drunk alcohol was greater among the 368 cases (83%) than among the 373 controls (69%). The estimated RR of breast cancer, with allowance for marital status only, was 2.2 (95% CI = 1.6–3.2). Allowance for all identified potential distorting factors did not appreciably modify the risk estimate (multivariate RR = 2.5, with 95% CI = 1.7–3.7).

Wine, of course, was the major source of alcohol in the population studied. When various levels of wine consumption were considered, a positive trend in risk was evident with increasing levels of exposure (test for trend: $\chi_1^2=23.6$, $P=0.001$), the point estimate increasing more than tenfold (with 95% lower confidence limit of about three) for more than half a liter/day. Likewise, combined use of more than one type of alcoholic beverage (wine/beer/spirits) gave an appreciable increase in the risk, the point estimates being 5.5 (univariate)

**Table II** Dist4ibution of 368 cases of breast cancer and 373 controls according to body mass index ($Kg m^{-2}$) and menopausal status. Pordenone, Italy, 1980–83.

| *Body mass index ($Kg m^{-2}$)* | *Breast cancer No.* | *(%)* | *Controls No.* | *(%)* | *M-H RR*[b] *(95% CI)* | *Multivariate RR*[c] *(95% CI)* |
|---|---|---|---|---|---|---|
| *Total* | | | | | | |
| <25 | 141 | (38) | 185 | (50) | (1)[a] | (1)[a] |
| 25–29 | 146 | (40) | 126 | (34) | 1.5 (1.1–2.1) | 1.4 (1.0–2.0) |
| ⩾30 | 77 | (21) | 56 | (15) | 1.9 (1.2–2.8) | 1.8 (1.1–2.8) |
| Unknown | 4 | (1) | 6 | (2) | — | — |
| *Pre-menopausal patients* | | | | | | |
| <25 | 51 | (57) | 83 | (65) | (1)[a] | (1)[a] |
| 25–29 | 30 | (34) | 28 | (22) | 1.7 (0.9–3.2) | 1.6 (0.8–3.3) |
| ⩾30 | 8 | (9) | 16 | (13) | 0.8 (0.3–2.1) | 1.3 (0.5–3.7) |
| *Post-menopausal patients* | | | | | | |
| <25 | 90 | (32) | 102 | (42) | (1)[a] | (1)[a] |
| 25–29 | 116 | (42) | 98 | (40) | 1.4 (0.9–2.0) | 1.4 (0.9–2.2) |
| ⩾30 | 69 | (25) | 40 | (16) | 2.1 (1.3–3.4) | 1.9 (1.1–3.3) |
| Unknown | 4 | (1) | 6 | (2) | — | — |

[a]Reference category.
[b]Adjusted for marital status.
[c]Adjusted for all identified potential distorting factors by means of multiple logistic regression.
Tests for linear trends:
- Total, Univariate: $\chi_1^2=10.81$, $P=0.001$; Multivariate: $\chi_1^2=6.63$, $P=0.01$.
- Pre-menopausal, Univariate: $\chi_1^2=0.22$, $P=0.64$; Multivariate: $\chi_1^2=1.03$, $P=0.31$.
- Post-menopausal, Univariate: $\chi_1^2=8.56$, $P=0.003$; Multivariate: $\chi_1^2=5.29$, $P=0.02$.

and 7.6 (multivariate), with lower 95% confidence limits higher than three. Drinking habits of cases and controls were compared within strata of age, geographical area, menopausal status, occupation, and parity; there was no evidence that the association was confined to any particular subgroup. Likewise, alcohol consumption was not appreciably different in controls with accidents or orthopaedic conditions, or other diseases.

### Discussion

The results of the present study suggest that a high socio-economic status, as indicated by years of education and occupation, was positively related to the risk of breast cancer. This finding agrees with previous reports (Kelsey, 1979). While the effect of education could be explained in terms of occupational categories, the effect of occupation was apparently independent of other socio-economic indicators (education and marital status), thus suggesting that some lifestyle habit, peculiar to specific occupational categories, may play a role. Among them, nutritional factors, specifically obesity and dietary factors, have received the most attention (Carroll, 1975; Miller *et al.*, 1978; Wynder, 1980; Lubin *et al.*, 1981; Graham *et al.*, 1982), and are supported by experimental animal models (Carroll, 1975; Welsch & Aylsworth, 1983).

In this study, a high body mass index was positively related with breast cancer risk. When separate analysis was made according to menopausal status, the association was apparently confined to postmenopausal women, in agreement with several previous studies (Mirra *et al.*, 1971;

**Table III** Distribution of 368 cases of breast cancer and 373 controls according to selected food items. Pordenone, Italy, 1980–1983.

| | *Breast cancer* No. | (%) | *Controls* No. | (%) | *M-H RR*[b] (95% CI) | *Multivariate RR*[c] (95% CI) |
|---|---|---|---|---|---|---|
| *Meat consumption* (days/week) | | | | | | |
| ⩾2 | 32 | (9) | 46 | (12) | (1)[a] | (1)[a] |
| 3–4 | 126 | (34) | 136 | (36) | 1.3 (0.8–2.2) | 1.1 (0.6–1.9) |
| ⩾5 | 210 | (57) | 191 | (51) | 1.6 (1.0–2.6) | 1.3 (0.7–2.2) |
| *Milk and dairy products* (days/week) | | | | | | |
| ⩽2 | 19 | (5) | 52 | (14) | (1)[a] | (1)[a] |
| 3–4 | 42 | (11) | 69 | (18) | 1.8 (0.9–3.4) | 1.5 (0.7–3.0) |
| ⩾5 | 307 | (83) | 252 | (68) | 3.4 (2.0–5.8) | 3.2 (1.8–5.8) |

[a]Reference category.
[b]Adjusted for marital status.
[c]Adjusted for all identified potential distorting factors by means of multiple logistic regression.
Test for linear trend:
– Meat consumption, Univariate: $\chi_1^2=3.91$, $P=0.05$; Multivariate: $\chi_1^2=1.2$, $P=0.27$.
– Milk and dairy products, Univariate: $\chi_1^2=27.42$, $P<0.001$; Multivariate: $\chi_1^2=23.79$, $P<0.001$.

**Table IV** Distribution of 368 cases of breast cancer and 373 controls according to various measures of alcoholic beverage consumption. Pordenone, Italy, 1980–83.

| | *Breast cancer* No. | (%) | *Controls* No. | (%) | *M-H RR*[b] (95% CI) | *Multivariate RR*[c] (95% CI) |
|---|---|---|---|---|---|---|
| *Total alcohol consumption* | | | | | | |
| Never | 62 | (17) | 116 | (31) | (1)[a] | (1)[a] |
| Ever | 306 | (83) | 257 | (69) | 2.2 (1.6–3.2) | 2.5 (1.7–3.7) |
| *Type of beverage consumption* | | | | | | |
| Never used | 62 | (17) | 116 | (31) | (1)[a] | (1)[a] |
| Wine only, beer only, or spirit only | 247 | (67) | 237 | (64) | 2.0 (1.4–2.8) | 2.3 (1.6–3.5) |
| Combined | 59 | (16) | 20 | (5) | 5.5 (3.1–9.7) | 7.6 (3.8–15.2) |
| *Current daily wine consumption* | | | | | | |
| Not used | 66 | (18) | 117 | (31) | (1)[a] | (1)[a] |
| ≦0.5 $l\ day^{-1}$ | 291 | (79) | 254 | (68) | 2.1 (1.5–2.9) | 2.4 (1.6–3.5) |
| >0.5 $l\ day^{-1}$ | 11 | (3) | 2 | (1) | 10.3 (2.8–38.0) | 16.7 (3.1–89.7) |

[a]Reference category.
[b]Adjusted for marital status.
[c]Adjusted for all identified potential distorting factors by means of multiple logistic regression.
Test for linear trend:
– Type of beverages, Univariate: $\chi_1^2=35.40$, $P<0.001$; Multivariate: $\chi_1^2=39.40$, $P<0.001$.
– Daily wine consumption, Univariate: $\chi_1^2=23.26$, $P<0.001$; Multivariate: $\chi_1^2=26.60$, $P<0.001$.

C

DeWaard & Halewijn, 1974; DeWaard, 1975; Kelsey, 1979; Helmrich *et al.*, 1983). This association is commonly explained by the higher levels of oestrogens in obese women, through accelerated peripheral aromatization of androstenedione to oestrone and, possibly, a greater availability due to a decrease in sex hormone binding globulin (Siiteri, 1978). The risk of breast cancer increased with the major indices of animal fat and animal protein intake considered (meat and dairy products), the trend being significant for dairy product intake. These results, too, are in line with previous reports (Miller *et al.*, 1978; Lubin *et al.*, 1981).

The major finding of the present study, however, lies in the positive association between breast cancer and alcohol consumption, women who drink alcoholic beverages displaying a relative risk more than double that of women who never drank. The estimated increase in the risk was greater for women who drank most frequently, and more than one type of alcoholic beverage.

It is unlikely that biased recall due to knowledge of the hypothesis explains this finding. At the time of data collection, the possible relation between breast cancer and alcohol consumption had not gained widespread attention in the lay press in Italy, and was almost certainly unknown to the interviewers and to the great majority of the subjects interviewed. Similarly, the possibility of systematic underreporting of alcohol consumption by non-neoplastic controls appears unlikely, as there is no widespread disapproval of alcohol consumption by women in this population. Furthermore, the proportion of drinkers was comparable in controls living within (70%) or outside the region (66%); married (70%) or unmarried (60%); nulliparous (67%) or parous (69%); less educated (<7 years, 67%) or more educated (69%); in pre- (69%) or post-menopause (69%); occupied in agriculture (75%) or in other occupations (68%). About two thirds of the control patients had been admitted following accidents, or on account of other orthopaedic conditions, which are reportedly positively associated with alcohol consumption. This possible bias, however, should lead, if anything, to an underestimate of the relative risks. With regard to confounding, the results were virtually unchanged when a large number of factors were taken into account.

Thus, our findings confirm the positive association between alcohol and breast cancer risk, originally reported by Rosenberg *et al.* (1982) in a study based on data from North America and Israel. Moreover, some selected information on dietary habits was available in the present study: when it was controlled for in the analysis, the relative risk estimates were not materially changed.

However, on an international level, alcohol is negatively correlated with breast cancer death rates (La Vecchia *et al.*, 1982). Moreover, the putative mechanism through which alcohol could exert a carcinogenic effect on breast cancer in humans is far from established. Increased prolactin secretion has been suggested, but the role of prolactin in the aetiology of breast cancer is not defined (Williams, 1976). Alternatively, alcohol-induced minor liver alterations might affect liver oestrogen metabolism, or the level of steroid binding globulins. Nevertheless, considering the broad heterogeneity of our population compared to the one studied by Rosenberg *et al.* (1982), (for instance, in this study alcohol was not positively correlated with education or smoking), the confirmation on our setting of the positive association between alcohol and breast cancer produces obvious evidence in favour of a role of alcohol consumption on the risk of breast cancer.

We are indebted to Ms. Angela Favot and Luisella Gottardi for interviewing cases and controls, and to Ms. J. Baggot and M.P. Bonifacino for editorial assistance.

Dr F. Colombo, from the "Mario Negri" Institute, Milan, assisted with data processing and computer programming; Dr G. Gallus, from the Institute of Medical Statistics, University of Milan, kindly commented the paper. Nurses and Clinicians of the Divisions of Medical Oncology, Orthopaedics, Dermatology, Surgery, and Clinical Medicine of the Ospedale Civile, Pordenone, Italy, helped in the identification and interviews of cases and controls.

This investigation was partly supported by C.N.R. (Italian National Research Council) grants for Epidemiological Surveillance of Oral Contraceptive Use Progetto Finalizzato "Medicina Preventiva – Contract no. 82.020.3856) and Clinical Pharmacology and Rare Diseases.

### References

ARMSTRONG, B. & DOLL, R. (1975). Environmental factors and cancer incidence and mortality in different countries, with special reference to dietary practices. *Int. J. Cancer,* **15,** 617.

BRESLOW, N.E. & DAY, N.E. (eds.) (1980). *Statistical Methods in Cancer Research,* IARC Scientific Publication, no. 32. IARC: Lyon.

CARROLL, K.K. (1975). Experimental evidence of dietary factors and hormone-dependent cancers. *Cancer Res.,* **35,** 3374.

DEWAARD, F. (1975). Breast cancer incidence and nutritional status with particular reference to body weight and height. *Cancer Res.,* **35,** 3351.

DeWAARD, F. & HALEWIJN, E.A.B. (1974). A prospective study in general practice on breast cancer risk in postmenopausal women. *Int. J. Cancer*, **14**, 153.

FLEISS, J. (1981). *Statistical Methods for Rates and Proportions*. New York: John Wiley, 2nd. ed.

GRAHAM, S., MARSHALL, J., METTLIN, C. & 3 others. (1982). Diet in the epidemiology of breast cancer. *Am. J. Epidemiol.*, **116**, 68.

HELMRICH, S.P., SHAPIRO, S., ROSENBERG, L. & 11 others. (1983). Risk factors for breast cancer. *Am. J. Epidemiol.*, **117**, 35.

KELSEY, J.L. (1979). A review of the epidemiology of human breast cancer. *Epidemiol. Rev.*, **1**, 74.

LA VECCHIA, C., FRANCESCHI, S. & CUZICK, J. (1982). Alcohol and breast cancer. *Lancet*, **i**, 621.

LEA, A.J. (1966). Dietary factors associated with death-rates from certain neoplasms in man. *Lancet*, **ii**, 332.

LUBIN, J.H., BURNS, P.E., BLOT, W.J. & 3 others. (1981). Dietary factors and breast cancer risk. *Int. J. Cancer*, **28**, 685.

MANTEL, N. (1963). Chi-square tests with one degree of freedom: Extension of the Mantel-Haenszel procedure. *J. Am. Stat. Assoc.*, **58**, 690.

MANTEL, N. & HAENSZEL, W. (1959). Statistical aspects of the analysis of data from retrospective studies of disease. *J. Natl Cancer Inst.*, **22**, 719.

MIETTINEN, O. (1976). Estimability and estimation in case-referent studies. *Am. J. Epidemiol.*, **103**, 226.

MILLER, A.B., KELLY, A., CHOI, N.W. & 7 others. (1978). A study of diet and breast cancer. *Am. J. Epidemiol.*, **107**, 499.

MIRRA, A.P., COLE, P. & MacMAHON, B. (1971). Breast cancer in an area of high parity, Sao Paulo, Brazil. *Cancer Res.*, **31**, 77.

REGIONE AUTONOMA FRIULI-VENEZIA GIULIA. (1973). *Compendio Statistico*. Friuli-Venezia Giulia.

ROSENBERG, L., SLONE, D., SHAPIRO, S. & 8 others. (1982). Breast cancer and alcoholic-beverage consumption. *Lancet*, **i**, 267.

SIITERI, P.K. (1978). Steroid hormones and endometrial cancer. *Cancer Res.*, **38**, 4360.

WELSCH, C.W. & AYLSWORTH, C.F. (1983). Enhancement of murine mammary tumorigenesis by feeding high levels of dietary fat: A hormonal mechanisms? *J. Natl Cancer Inst.*, **70**, 215.

WILLIAMS, R.R. (1976). Breast and thyroid cancer and malignant melanoma promoted by alcohol-induced pituitary secretion of prolactin, TSH, and MSH. *Lancet*, **i**, 996.

WYNDER, E.L. (1980). Dietary factors related to breast cancer. *Cancer*, **46**, 899.